# Mickey Mouse Sign on Bone Scan in the Monostotic Form of Paget’s Disease Mimicking Osseous Metastasis

**DOI:** 10.4274/mirt.galenos.2019.75437

**Published:** 2020-10-19

**Authors:** Selin Kesim, Halil Turgut Turoğlu, Salih Özgüven, Tunç Öneş, Tanju Yusuf Erdil

**Affiliations:** 1 Marmara University Training and Research Hospital, Clinic of Nuclear Medicine, İstanbul, Turkey

**Keywords:** Paget’s disease, monostotic Paget’s disease, spine, Mickey Mouse sign, technetium 99m-methylene diphosphonate bone scintigraphy, SPECT/CT

## Abstract

Paget’s disease is a chronic benign bone disease characterized by excessive and abnormal bone remodeling. Monostotic Paget’s disease accounts for only 20% of the cases, and the monostotic form involving the vertebra with the Mickey Mouse sign is very rare. Herein, we report a case of suspected bony metastasis in the second lumbar vertebra that was diagnosed as Paget’s disease because of the Mickey Mouse sign on bone scintigraphy, and the diagnosis was confirmed by biopsy. Therefore, bone scintigraphy may provide a positive contribution to the diagnosis, and may help to avoid unnecessary biopsy in cases with specific signs and patterns.

## Abstract (tr)

Paget, aşırı ve anormal kemik yapımı ve yıkımı ile karakterize kronik, iyi huylu bir kemik hastalığıdır. Monostotik Paget hastalığı, sadece olguların %20’sini oluşturmakla kalmaz; Mickey Mouse bulgusu olan, vertebra tutulumu ile giden monostotik formu daha da nadirdir. Sunduğumuz olguda, ikinci lumbar vertebrada metastaz şüphesi ile araştırılan hastada; kemik sintigrafisinde Mickey Mouse işareti görülmesi üzerine Paget hastalığı tanısı kondu ve tanı biyopsiyle doğrulandı. Bu olguda görülmektedir ki; kemik sintigrafisi tanıya olumlu katkıda bulunur ve belirli belirti ve paternler görülmesi sayesinde gereksiz biyopsileri önleyebilir.

## Figures and Tables

**Figure 1:**
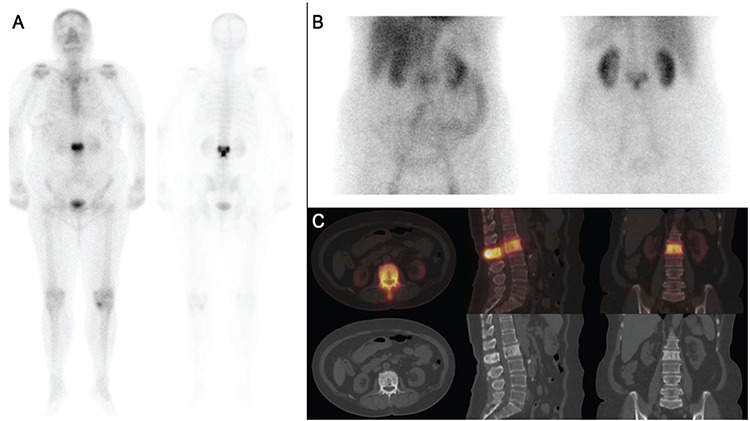
A 62-year-old woman who presented with a two-year history of low back pain was referred for bone scintigraphy. Technetium 99m-methylene diphosphonate [Tc-99m methyl diphosphonate (MDP)] whole body bone scan revealed an intensely increased uptake throughout the whole second lumbar vertebra (L-2), involving the body, posterior elements, and spinous process (A). The simultaneously performed three-phase bone scintigraphy demonstrated increased blood flow and local hyperemia on the respective dynamic blood flow and static blood pool images that accompanied the increased uptake involving the L-2 vertebra (B). The single photon emission computerized tomography/computed tomography (SPECT/CT) hybrid imaging revealed vertebral expansion with diffuse sclerosis involving the body and posterior vertebral arch of the second lumbar vertebra, correlating with the diffuse and intense Tc-99m MDP uptake in the entire vertebra (C). Paget’s disease of bone (PD), which is a chronic benign bone disease characterized by excessive and abnormal bone remodeling, has three phases: the early lytic phase, the second mixed phase, and the final sclerotic phase. The pelvis is the most commonly affected bone, followed by the spine, skull, femur, scapula, tibia, and humerus. Pagetic lesions are commonly (approximately 70-80% of the cases) multiple (polyostotic) lesions (1). Bone scintigraphy is useful not only to survey the entire skeleton for PD, but also to screen for complications like fracture and malignant transformation, and to monitor the response to therapy. The monostotic form of PD, as in this case, is not common and may lead to a misdiagnosis with a variety of metabolic and neoplastic diseases (2). Vertebral neoplasia (including metastases) may involve both the vertebral body and partially the posterior vertebral arch. However, the spinous process is spared in most of these cases. Multimodality imaging using s SPECT/CT integrates different techniques to make a correct diagnosis, and avoids unnecessary biopsy. The advantages of bone SPECT/CT imaging are as follows: it may be performed on the same day after the bone scan and it provides anatomical detail and morphological information (3,4,5). The “Mickey Mouse” or “Mouse Face” sign, which typically shows an upside-down triangle consisting of three foci of intense radiopharmaceutical uptake, and corresponding to the involvement of the pedicles and spinous process, is a specific and rare pattern of Paget’s disease. This sign was originally described by Van Heerden (6). Subsequently, Kim et al. (7) reported this “Mouse Face” appearance of the vertebrae as a specific finding of bone PD. Additionally, Rotés-Sala et al. (8) described the “clover sign,” where the vertebral pedicles and spinous process are affected. The recognition of typical patterns like the “Mickey Mouse sign” in this case, together with the increased blood flow and hyperemia on the three-phase bone scintigraphy and using the multimodality imaging (SPECT/CT in our case) to demonstrate the accompanying vertebral expansion, and sclerosis increases the specificity and diagnostic accuracy of bone scan in the identification of PD

**Figure 2:**
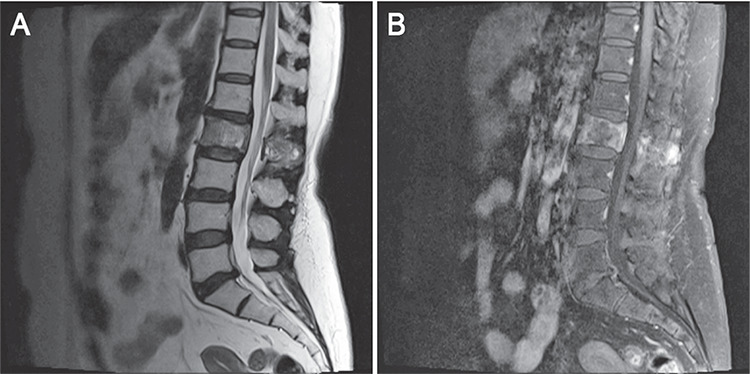
T2-weighted lumbar magnetic resonance imaging (MRI) images depicted diffuse heterogeneous density changes (A) and contrast-enhanced T1-weighted MRI images showed contrast enhancement involving the body of the second lumbar vertebra that was extending to the posterior elements, and reported as suspicion for osseous metastasis (B)
